# ZnO-Based Electrochemical Immunosensor to Assess Vaccine-Induced Antibody-Mediated Immunity against Wild-Type and Gamma SARS-CoV-2 Strains

**DOI:** 10.3390/bios13030371

**Published:** 2023-03-11

**Authors:** Freddy A. Nunez, Ana C. H. Castro, Isabela P. Daher, Edecio Cunha-Neto, Jorge Kalil, Silvia B. Boscardin, Alexandre J. C. Lanfredi, Vivian L. de Oliveira, Wendel A. Alves

**Affiliations:** 1 Centro de Ciências Naturais e Humanas, Universidade Federal do ABC, São Paulo 09210-580, Brazil; 2 Laboratorio de Imunologia, INCOR, Hospital das Clinicas HCFMUSP, Faculdade de Medicina, Universidade de Sao Paulo, São Paulo 05403-900, Brazil; 3 Departamento de Parasitologia, Instituto de Ciências Biomédicas, Universidade de Sao Paulo, São Paulo 05508-900, Brazil; 4 LIM-19, Hospital das Clinicas HCFMUSP, Faculdade de Medicina, Universidade de Sao Paulo, São Paulo 05403-900, Brazil; 5 Centro de Engenharia, Modelagem e Ciências Sociais Aplicadas, Universidade Federal do ABC, São Paulo 09210-580, Brazil

**Keywords:** SARS-CoV-2, electrochemical immunosensor, zinc oxide nanorods, serological assessment, antibody-mediated immunity, COVID-19 vaccines, ChAdOx1-S (Oxford–AstraZeneca), BNT162b2 (Pfizer–BioNTech)

## Abstract

The evaluation of serological responses to COVID-19 is crucial for population-level surveillance, developing new vaccines, and evaluating the efficacy of different immunization programs. Research and development of point-of-care test technologies remain essential to improving immunity assessment, especially for SARS-CoV-2 variants that partially evade vaccine-induced immune responses. In this work, an impedimetric biosensor based on the immobilization of the recombinant trimeric wild-type spike protein (S protein) on zinc oxide nanorods (ZnONRs) was employed for serological evaluation. We successfully assessed its applicability using serum samples from spike-based COVID-19 vaccines: ChAdOx1-S (Oxford–AstraZeneca) and BNT162b2 (Pfizer–BioNTech). Overall, the ZnONRs/ spike-modified electrode displayed accurate results for both vaccines, showing excellent potential as a tool for assessing and monitoring seroprevalence in the population. A refined outcome of this technology was achieved when the ZnO immunosensor was functionalized with the S protein from the P.1 linage (Gamma variant). Serological responses against samples from vaccinated individuals were acquired with excellent performance. Following studies based on traditional serological tests, the ZnONRs/spike immunosensor data reveal that ChAdOx1-S vaccinated individuals present significantly less antibody-mediated immunity against the Gamma variant than the BNT162b2 vaccine, highlighting the great potential of this point-of-care technology for evaluating vaccine-induced humoral immunity against different SARS-CoV-2 strains.

## 1. Introduction

The prolongation of the COVID-19 pandemic is mainly due to the emergence of variants of SARS-CoV-2. Mutations acquired by the virus, which lead to the emergence of new variants, influence disease severity as well as the performance of current COVID-19 vaccines or therapeutic drugs and raise questions about the effectiveness of the available diagnostic tools [1].

Beyond the current public health challenge of vaccine coverage against SARS-CoV-2 new variants, the development of better testing tools for the assessment of vaccine-based population immunity is essential to overcoming the COVID-19 pandemic. Investments in research and development of vaccine-induced immunity evaluation tools are crucial to improving the effectiveness and sustainability of vaccination campaigns, especially in the current context of SARS-CoV-2 new variants that continue to spread rapidly and evade vaccine-induced immune responses despite the high vaccine coverage.

Mutations arise as a natural consequence of viral replication; RNA viruses naturally have higher mutation rates than DNA viruses [2]. Viral mutations are also influenced by the selective pressure induced by mass vaccination with S protein-based vaccines. Generating focal polyclonal immune responses targeting a single antigen is a selective pressure on the immune system that favors, for example, the induction of mutation hotspots in the RBD (receptor-binding domain) region of the viral spike protein and may contribute to the perpetuation of the COVID-19 pandemic [3,4]. Spike protein has been broadly explored as the primary protein target in the development of the COVID-19 vaccine and diagnostic antigen-based testing. It is required for virus infectivity, participating in the mechanisms of virus binding, fusion, and entry into host cells [1,2]. Furthermore, S protein is considered the main antigenic element among the structural viral proteins, inducing host immune responses and potent neutralizing antibodies.

Among the main vaccines focused on inducing anti-S protein immune responses currently used worldwide, we can cite ChAdOx1-S (Oxford–AstraZeneca) and BNT162b2 (Pfizer–BioNTech). ChAdOx1-S consists of the replication-deficient simian adenoviral vector used to deliver the full-length SARS-CoV-2 structural S protein as a vaccine [5]. BNT162b2 is a lipid nanoparticle-formulated, nucleoside-modified mRNA encoding the SARS-CoV-2 S protein stabilized in its prefusion conformation [6]. Even though mass vaccination worldwide has successfully restrained the COVID-19 pandemic, its performance against variants and its potential role in inducing selective evolutionary pressure favoring the emergence of novel variants remain under discussion. For these reasons, it is crucial to develop mass testing tools to measure the vaccines’ efficacy against SARS-CoV-2 variants that would allow us to better understand vaccine-based population immunity to further optimize public health assistance and vaccination programs, as well as to support future individualized vaccine protocols.

Since the beginning of the COVID-19 pandemic, numerous tests have been developed to detect anti-SARS-CoV-2 antibodies [7,8,9,10]. Schasfoort et al. describe a test based on surface plasmon resonance (SPR) to determine the presence and binding force of IgG, IgM, and IgA antibodies against the receptor binding domain (RBD) of the S protein. They evaluated the binding force during the disease, observing an increase in IgG levels and binding force while IgM and IgA levels decreased. This assay provides information on the immunological status of patients [11]. Huang et al. developed a biosensor by combining nanoplasmonic immunosorbent with nanoporous hollow gold nanoparticles to improve sensitivity due to the increased SPR effect, achieving a detection limit of 0.2 pm within 15 min, and used the system to quantify SARS-CoV-2 neutralizing antibodies in individual post-vaccination serum samples [12]. Gong et al. presented a solid-state electroluminescence platform using a silica nano-channel matrix, cationic [Ru(bpy)_3_]^2+^, and the S protein to detect SARS-CoV-2 antibodies, with a detection limit of 2.9 pg mL^−1^ within 30 min of incubation [13]. Rahmati et al. developed an electrochemical immunosensor to analyze SARS-CoV-2 antibodies. They used screen-printed carbon electrodes modified with nickel nanoparticles, functionalized with the S protein, and blocked with bovine serum albumin (BSA) to decrease nonspecific binding, achieving a detection limit of 0.3 fg mL^−1^ within 20 min [14]. Despite being innovative and sophisticated detection systems compared to electrochemical sensing techniques, they are not yet so attractive commercially. So far, electrochemical biosensors have been widely explored because they are point-of-care test systems with rapid, accurate detection, low production costs, and the ability to require small samples for detecting and analyzing results [15].

Previous work by our group [16] demonstrated the successful development and validation of a serological point-of-care ZnONRs/spike immunosensor to detect anti-SARS-CoV-2 antibodies in individuals immunized with an inactivated COVID-19 vaccine (SinoVac–CoronaVac). The electrochemical immunosensor was developed with the immobilization of the recombinant trimeric S protein by zinc oxide nanorods (ZnONRs) over the chemically modified fluorine-doped tin oxide (FTO) substrate. In brief, the wild-type S protein was employed as the biorecognition element onto the modified ZnONRs surface. Then, when serum containing anti-SARS-CoV-2 antibodies is applied, anti-S antibodies form antigen-antibody complexes with the S protein along the electroconductive surface, and an electrochemical signal is generated. In this way, the immunosensor produces a measurable and proportional electrical signal. Processing and amplification of the signal are performed by an electronic circuit, allowing for a graphical representation of the result. The immunosensor is easy to produce and may be combined with any handheld computer. In addition, it holds great potential for other diagnostic applications since the architecture of the sensor technology can be easily recreated, setting up a custom-made variation using different biomolecules onto the ZnONRs.

The current study highlights the applicability of a ZnO-based electrochemical immunosensor, exploring the impedimetric biosensor tool for the assessment of humoral immunity after a second dose of the spike-based COVID-19 vaccines, ChAdOx1-S (Oxford–AstraZeneca) and BNT162b2 (Pfizer–BioNTech). In addition, both S protein-based vaccines were evaluated and showed excellent performance in detecting anti-SARS-CoV-2 antibodies against the wild-type S protein. Our experimental data prove that this biosensor architecture is modular and adaptable to evaluate antibody-mediated immunity against another highly transmissible SARS-CoV-2 strain, the Gamma coronavirus variant, also called the P.1 strain, one of the most virulent strains, which appears to have evolved and devastated the city of Manaus, the capital of Amazon State in Brazil, causing a deadly second wave of infections in early 2021. It is worth noting that the assessment of vaccine-elicited immunity by the FTO-ZnONRs/spike WT immunosensor showed greater titres of anti-S antibodies in serum samples from vaccinated study participants of both vaccines analyzed, ChAdOx1-S (Oxford–AstraZeneca) and BNT162b2 (Pfizer–BioNTech), when compared to the previously published study from our group, based on serum samples from study participants vaccinated with the inactivated virus-based CoronaVac vaccine [16].

The sensor is able to detect vaccine-induced antibody responses against different variants of SARS-CoV-2 that are probably cross-reactive. In this way, it consists of a COVID-19 serological point-of-care test dedicated to assessing the levels of anti-S antibodies. We have already shown that the sensor works very well when the WT S protein is used to detect the presence of antibodies induced by vaccination with CoronaVac [16]. Now we show that, when the sensor is adapted with another biorecognition element (the spike protein derived from the P.1 (Gamma variant)), it is also able to detect the anti-S antibody response elicited in individuals previously vaccinated with ChAdOx1-S (Oxford–AstraZeneca) and BNT162b2 (Pfizer–BioNTech) vaccines. Therefore, the highlight of this paper is the demonstration that the technology we developed is adaptable and presents an excellent performance in detecting antibody responses induced after vaccination, even when we use the S protein derived from a different strain as the P.1 (Gamma variant). This feature makes the point-of-care device more attractive for use in the new public health landscape, where a large portion of the population is subject to different vaccination schemes and epidemiological contexts.

The simple replacement of the bioreceptor by the correspondent mutated recombinant S protein, immobilized on the surface of the adapted ZnO-based electrochemical immunosensor, enabled the accurate discrimination of different serological vaccine-induced immunity profiles via electrochemical signals by either the mRNA or adenovirus vector spike-based vaccines tested. These findings align with previous data concerning vaccine-induced immunity using conventional serological detection methods [17], confirming that our biosensing platform is in line with real-world data and is a high-performance point-of-care serology test for COVID-19. As a fast and inexpensive device, it could be used in the future as a rapid serological test to customize COVID-19 vaccine booster recommendations based on individual vaccine-induced humoral responses.

## 2. Materials and Methods

### 2.1. Materials

The following reagents were purchased from Sigma-Aldrich (São Paulo, Brazil): zinc nitrate hexahydrate (reagent grade: 98%-cat# 228737), hexamethylenetetramine (ACS reagent: ≥99%-cat# 398160), glycine (ACS reagent: ≥98.5%-cat# 410225), potassium ferricyanide (ACS reagent: ≥99%-cat# P8131), and potassium ferrocyanide (ACS reagent: 98.5%-cat# P9387). Potassium chloride (reagent grade: 99%-cat# C2010.01.AG) was purchased from Labsynth (São Paulo, Brazil). The SARS-CoV-2 recombinant trimeric spike glycoprotein from the wild-type strain was kindly donated by the Cell Culture Engineering Laboratory (LECC) of COPPE/Federal University of Rio de Janeiro (UFRJ-Brazil). The SARS-CoV-2 recombinant spike glycoprotein from SARS-related coronavirus 2, P.1 lineage (cat# NR-55307) and the monoclonal anti-SARS-CoV-2 S protein-similar to 240C (cat# NR-616) were donated by BEI Resources.

### 2.2. Human Serum Samples

The seronegative control samples provided by the Cell Bank (BCRJ) were included in the study through the approved Ethics Committee of the Federal University of ABC (CAAE: 43139921.2.0000.5594). These control serum samples were collected from venous blood before the COVID-19 pandemic (*n* = 10). The ChAdOx1-S (Oxford–AstraZeneca) (*n* = 29) and BNT162b2 (Pfizer–BioNTech) (*n* = 29) venous blood were collected from vaccinated study participants who reported no previous infection with SARS-CoV-2 at least 28 days after the second immunization. Written informed consent was approved by the Ethics Committee in Research of the Clinics Hospital of the University of Sao Paulo Medical School (HC-FMUSP CAPPesq-CAAE: 30155220.3.0000.0068) and signed by all vaccinated study participants. Samples were categorized along with the article as pre-pandemic, AstraZeneca WT (serum from individuals vaccinated with ChAdOx1-S and evaluated using the spike WT protein as antigen), Pfizer WT (serum from individuals vaccinated with BNT162b2 and evaluated using the spike WT protein as antigen), AstraZeneca P.1 (serum from individuals vaccinated with ChAdOx1-S and evaluated using the spike P.1 protein as antigen), and Pfizer P.1 (serum from individuals vaccinated with BNT162b2 and evaluated using the spike P.1 protein as antigen).

### 2.3. Biosensor Construction

**Figure 1:**
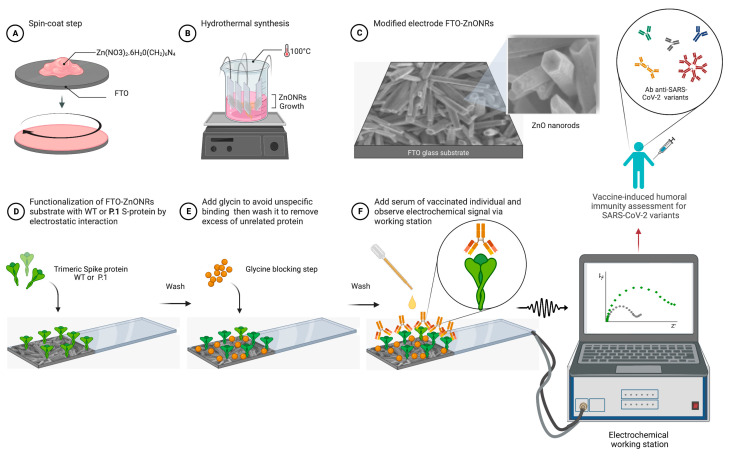
Schematic processes for the FTO-ZnONRs spike-based sensing immunosensor platform’s fabrication. Step-by-step process of the immunosensor fabrication, including (**A**) Spin-coat procedure, (**B**) Hydrotermal synthesis, (**C**) Modified electrode FTO-ZnONRs, (**D**) Functionalization of FTO-ZnONRs substrate with S proteins, (**E**) Blocking step with glycine, and (**F**) Human serum incubation followed by the acquisition of the electrochemical signal through an electrochemical working station for humoral immunity assessment. Created with BioRender.com.

Fluorine-doped and electrochemical sensing layers were performed as previously described by our group [16]. In brief, solutions of Zn(NO_3_)_2_ (20 µL, 0.5 mol L^−1^) and HMT (20 µL, 0.5 mol L^−1^) were applied onto the FTO substrate and spun at a controlled speed to create a thin and organic homogeneous film (Figure 1A). The ZnONRs films were grown hydrothermally by soaking the modified substrates in a mixture of 1:1 (*v*/*v*) of Zn(NO_3_)_2_ (0.1 mol L^−1^) and HMT (0.1 mol L^−1^) (Figure 1B). Hydrothermal growth was performed at 100 °C for 4 h in a sealed beaker to generate the modified electrode called FTO-ZnONRs (Figure 1C). Next, the FTO-ZnONRs electrode was rinsed with phosphate-buffer saline (PBS) to generate a hydrophilic surface; then, a drop containing the volume of 20 μL of SARS-CoV-2 recombinant spike protein from WT or P.1 strains, 4 μg mL^−1^ diluted in PBS (pH 7.4) was applied onto the FTO-ZnONRs surface and left incubated for 5 h for adsorption (Figure 1D). Then ZnONRs surface was rinsed to remove any unbound recombinant protein and dried. To reduce non-specific binding, glycine solution (10 μmol L^−1^) was added; a volume of 20 μL was placed and left incubating for 30 min, and the device was again washed and dried (Figure 1E). Glycine was used as an agent to prevent non-specific antibody binding on the device surface and to have a low dielectric constant [18,19,20,21]. In previous work, we have demonstrated that the glycine coating works directly on the ZnO nanorods since it has a good affinity for carboxylic groups [22]. A volume of 20 μL of serum sample diluted 1:500 (*v*/*v*) in PBS (pH 7.4) was incubated for 1 h on the electrode surface, rinsed to wash away the unbound material (non-specific antibodies), and subsequently dried. Finally, the electrochemical signal is acquired through an electrochemical working station for humoral immunity assessment (Figure 1F).

### 2.4. Morphological and Spectroscopic Characterizations

An ultra-high vacuum field emission scanning electron microscope (FESEM) for high-resolution imaging, JMS-6701F (JEOL, Tokyo, Japan) was used to obtain the SEM images at 5 kV of voltage. The Fourier transform infrared analysis was recorded using 124 scans between 700 and 1800 cm^−1^ at a resolution of 4 cm^−1^.

### 2.5. Electrochemical Measurements

The electrochemical data were collected using a μAutolab type III/FRA2 potentiostat/galvanostat, using the NOVA software version 2.1.3 supplied by Metrohm. In a traditional three-electrode system, an FTO-ZnONRs-modified electrode was used as a working electrode, Pt as a counter electrode, and SCE as a reference electrode. These electrochemical experiments were carried out in 5 mmol L^−1^ K_4_Fe(CN)_6_/K_3_Fe(CN)_6_ in 0.1 mol L^−1^ KCl (pH 7.3). The EIS data were analyzed using the modified Randles circuit model.

### 2.6. Enzyme-Linked Immunosorbent Assay (ELISA) Assay

ELISA was performed for the detection of IgG antibodies against the WT S protein expressed in Expi293F^TM^ cells (ThermoFisher, Waltham, MA, USA). Briefly, 96-well high-binding ELISA plates (Corning, New York, NY, USA, EUA) were coated with 50 µL of 100 ng/well of WT S protein diluted in PBS overnight at room temperature. Coat solution was discarded, and the plates were washed five times with wash solution (PBS-Tween 20 0.05%) and incubated with 150 µL of blocking buffer (PBS-Tween 20 0.02%, non-fat milk 5%, and BSA 1%) for 1 h at room temperature. Plates were washed 5 times, as previously described, and serum was diluted in dilution buffer (PBS-Tween 20 0.02%, non-fat milk 5%, and BSA 0.25%) at 1:250 for the ChAdOx1-S (Oxford–AstraZeneca), 1:500 for the BNT162b2 (Pfizer–BioNTech), and 1:250 and 1:500 for the pre-pandemic sera. Then, 100 µL of the dilution was added to each well and incubated for 2 h at room temperature. After another round of plate washes (5 times), 50 µL of the secondary antibody goat anti-human IgG-HRP (1:15,000, KPL) were added to the plates for 1 h at room temperature. Plates were vigorously washed five times, and the enzymatic reaction was developed by the addition of 1 mg/mL of ortho-phenylenediamine dihydrochloride (OPD) (Sigma-Aldrich, Saint Luis, EUA) diluted in phosphate–citrate buffer, pH 5.0, containing 30% (*v*/*v*) hydrogen peroxide (Merck KGaA, Darmstadt, Germany). Then, 100 µL of the final solution was added to each well, and the plates were incubated for 15 min at room temperature in the dark. Reaction was stopped using 50 µL of sulfuric acid 4N. OD_492nm_ was measured using a microplate reader (BioTek, Winooski, USA). Results are given as the average of OD_492nm_ from each sample.

### 2.7. Statistical Analysis

Kruskal–Wallis test with Dunn’s post-hoc test for multiple comparisons was used to analyze the variables. Receiver operating characteristics (ROCs) analysis was used to verify the performance of the sensors ZnONRs/spike WT and the ZnONRs/spike P.1 immunosensors. The Spearman rank correlation coefficient was used to determine the correlation between the ZnONRs/spike WT results and P.1 immunosensors. Experimental data analysis was performed using GraphPad Prism software version 9, and the result of *p* < 0.05 was considered statistically significant.

## 3. Results and Discussion

### 3.1. Detection of Anti-S Protein Antibodies from Individuals Vaccinated with ChAdOx1-S (Oxford–AstraZeneca) and BNT162b2 (Pfizer–BioNTech) Using the FTO-ZnONRs/Spike WT Immunosensor

To further explore the applicability of the developed ZnO electrochemical immunosensor to detect antibodies against the wild-type spike protein [16], we evaluated the induced anti-S antibody responses of 29 serum samples from individuals vaccinated with ChAdOx1-S (Oxford–AstraZeneca), an adenovirus-based vaccine, and 29 serum samples from individuals vaccinated with BNT162b2 (Pfizer–BioNTech), an mRNA vaccine, using the electrochemical impedance spectroscopy (EIS) technique. Impedance variations come from changes in the physical interfacial properties since most of the biological molecules adsorbed by the electrochemical surface have an insulating nature, promoting increased electrical impedance [23,24].

**Figure 2:**
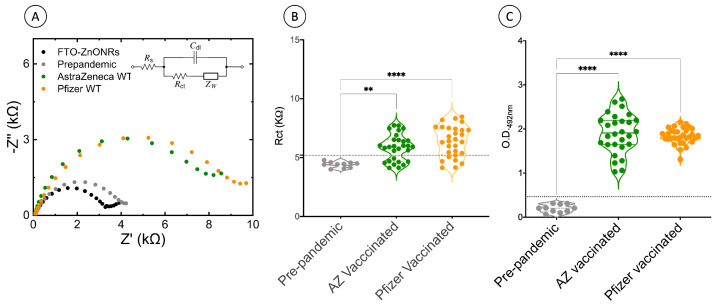
Vaccine-induced antibody-mediated immunity assessment by the FTO-ZnONRs/spike wild-type immunosensor. (**A**) Nyquist diagram of EIS measurements of serum samples from pre-pandemic, ChAdOx1-S (Oxford-AstraZeneca), BNT162b2 (Pfizer–BioNtech) vaccinated individuals, and, as an internal control, the FTO-ZnONRs electrode without serum samples. Data were acquired with the following settings: 10 mV of RMS amplitude and a range of 30 kHz to 0.1 Hz. Inset: the equivalent circuit used for electrochemical impedance data acquisition. (**B**) Immunosensor detection of antibody responses against S protein of the SARS-CoV-2 wild-type strain (WT spike) of a representative collection of serum samples from healthy pre-pandemic and ChAdOx1-S (Oxford–AstraZeneca) and BNT162b2 (Pfizer–BioNtech) vaccinated individuals. The electrochemical impedance method was used for all sample acquisitions, and data were represented as charge transfer resistance (R_ct_). The cutoff value was established as the average R_ct_ values obtained from the analysis of all negative sera within three standard deviations. Electrolyte: 5 mmol L^−1^ K_4_Fe(CN)_6_/K_3_Fe(CN)_6_ solution in 0.1 mol L^−1^ KCl. (**C**) ELISA anti-S results measurements of the same serum samples. Results are represented as OD_492nm_. Violin plots, where symbols represent individual participants. Asterisks indicate significant results ** *p* < 0.01 and **** *p* < 0.0001 using the Kruskal–Wallis test with Dunn’s post-hoc test for multiple comparisons. AZ = ChAdOx1-S (AstraZeneca). Pfizer = BNT162b2 (Pfizer–BioNTech).

The FTO-ZnONRs/spike WT immunosensor read-outs (R_ct_ signal) from ChAdOx1-S and BNT162b2 vaccinated individuals showed significantly higher R_ct_ values than the CoronaVac vaccinated serum samples analyzed in our previous work [16]. We also observed an increase in the R_ct_ signal (Figure 2A,B) in the serum samples from BNT162b2 compared to ChAdOx1-S vaccinated individuals, indicating more antibodies were present in these samples. Higher antibody titers were previously reported in individuals vaccinated with BNT162b2 compared to those immunized with the ChAdOx1-S vaccine [25]. The ELISA results demonstrated that the immunization protocols employed with both vaccines induced comparable levels of anti-spike IgG antibodies (Figure 2C). The IgG levels in both groups were significantly higher compared to the unvaccinated pre-pandemic controls. In this way, we have demonstrated the validation of the immunosensor with the gold standard methodology for assessment of humoral immune responses against COVID-19 using samples from vaccinated individuals with the most commonly used spike protein-based vaccines.

Our sensor-based data also agree with previous studies using conventional serological/ELISA tests, where the administration of both tested vaccines induced higher titers of antibodies against the S protein than the inactivated SARS-CoV-2 vaccine, CoronaVac [26,27]. This behavior is expected since both vaccines have been developed to elicit antibodies against the S protein of SARS-CoV-2 but not against other viral proteins, as in the CoronaVac vaccine. Indeed, we have also demonstrated lower levels of anti-S protein antibodies in our previous study with CoronaVac-vaccinated samples [12]. Electrochemical data read-out variations across individuals observed in this study are also expected since different biotechnological approaches to presenting the S protein in the vaccine formulation to the immune system were employed in each vaccine. Furthermore, it is well known that immunity depends on various intrinsic host factors (such as age, gender, ethnicity, and comorbidities) and external factors (such as preexisting immunity, environment, and behavior) [28].

### 3.2. Antibody Responses Using the Adapted P.1 Spike ZnONRs Immunosensor

#### 3.2.1. Morphological and Spectroscopic Characterization

**Figure 3:**
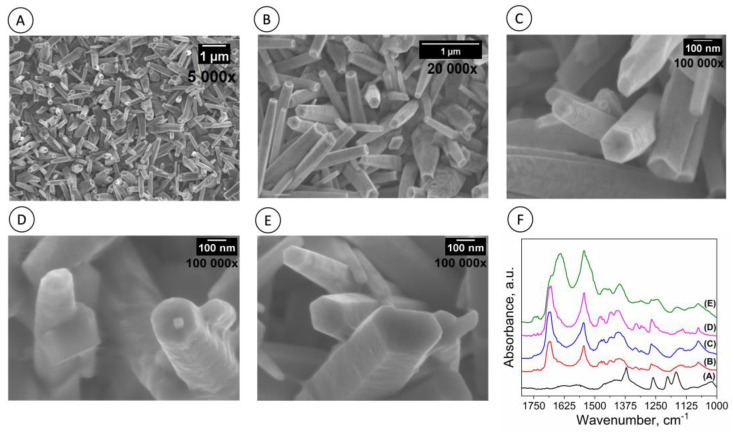
Scanning Electron Microscopy (SEM) images and FTIR spectra of the ZnONRs immunosensor surface at different stages of fabrication. (**A**–**C**) Representative SEM images showing the morphology of the ZnONRs without any modification. (**D**) Representative SEM image shows the surface of the ZnONRs after the adsorption of spike WT protein. (**E**) Representative SEM image shows the surface after spike P.1 protein adsorption. (**F**) FTIR spectra at each stage of electrode fabrication: Chart label (**A**) FTO-ZnONRs, (**B**) FTO-ZnONRs/spike, (**C**) FTO-ZnONRs/spike/gly, (**D**) FTO-ZnONRs/spike/gly/negative serum samples (pre-pandemic samples collected from healthy donors), and (**E**) FTO-ZnONRs/spike/gly/positive serum samples (samples collected from vaccinated individuals). Up right labels indicate scale bars and corresponded image magnification (×).

The SEM images in Figure 3A,B demonstrate that ZnONRs are highly dense and grown on the surface of FTO glass substrates. The high-magnification SEM image in Figure 3C reveals that the ZnONRs obtained possess a hexagonal morphology, with an average diameter of approximately 200–300 nm, consistent with results reported elsewhere [16,22,29]. SEM images of the FTO-ZnONRs/spike WT and the FTO-ZnONRs/spike P.1 electrodes are presented in Figure 3D,E, respectively. Subtle differences can be observed in both modified electrodes when compared to the bare ZnONRs electrode. Specifically, the surface of the nanorods modified with proteins appears rougher and less smooth than the FTO-ZnONRs electrode (Figure 3C), which could indicate successful functionalization with the S protein. Furthermore, due to the large surface area of the modified electrodes, they are expected to enhance charge transfer efficiency and sensing properties.

The FTIR analysis (Figure 3F) confirmed the attachment of the S protein P.1 to the electrode surface, as evidenced by the characteristic bands of amide-I, II, and III, which is consistent with previous findings [16]. Particularly, when convalescent serum was tested, significant increases in bands associated with IgG, IgM, and IgA were observed at 1560–1464 cm^−1^, 1420–1289 cm^−1^, 1160–1028 cm^−1^, and 1285–1237 cm^−1^, respectively [30]. These bands were broad and overlapped on the spectrum of the spike-immobilized electrode surface, indicating that this surface could be used to detect the anti-S antibodies.

#### 3.2.2. Calibration Curve of the FTO-ZnONRS/Spike P.1 (Gamma Variant) Immunosensor

**Figure 4:**
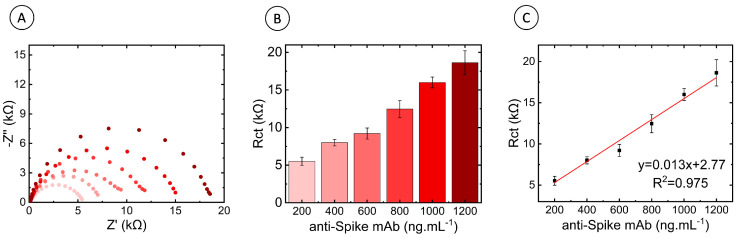
Anti-spike monoclonal antibody binding studies using FTO-ZnONRs/spike P.1 (Gamma variant) electrode. R_ct_ values of the calibration study varying the concentration of the anti-S protein monoclonal antibody. (**A**) Nyquist diagram and (**B**) Histogram showing the impedance values obtained. The error bars correspond to the standard deviation of the data points. Each concentration label matches the corresponding line colors in graph A, (**C**) Calibration curve. Electrolyte: 5 mmol L ^−1^ K_4_Fe(CN)_6_/K_3_Fe(CN)_6_ solution in 0.1 mol L ^−1^ KCl.

Calibration studies using an anti-S monoclonal antibody (mAb) were performed to set up the spike P.1 (Gamma variant) immunosensor and ensure a measurable and specific proportional electrical signal. The calibration curve was made to measure the immunosensor electrochemical behavior response to the binding detection of the anti-S mAb (anti-SARS-CoV-2 spike protein—similar to 240C) in the concentration range of 200 to 1200 ng·mL^−1^. Figure 4A shows the EIS plots obtained after mAb incubation, and the results show that the semicircle radius increased with the increase in mAb concentration. Figure 4B shows the histogram of the EIS measurements for each concentration, calculated from three individual immunosensors. The R_ct_ values were plotted against the mAb concentration (Figure 4C). The analytical curve showed a good linear regression (R^2^ = 0.975), a limit of detection (LOD) of 52.55 ng·mL^−1^ for mAb detection, and a limit of quantification (LOQ) of 159.23 ng·mL^−1^. Our previous studies (carried out with the spike WT immunosensor) [16] obtained a LOD of 19.34 ng·mL^−1^ for mAb detection and a LOQ of 58.62 ng·mL^−1^. This performance demonstrates that the immunosensor is suitable for detecting anti-SARS-CoV-2 spike WT and anti-SARS-CoV-2 spike P.1 (Gamma variant) antibodies. The determined LOD values are comparable with other immunoassays and, in some cases, show higher sensitivity (Table 1). Nevertheless, the reported device is easier to fabricate, cheaper, and portable, factors that emphasize its utility.

#### 3.2.3. Detection of Antibody Responses from Vaccinated Individuals Using the P.1 Strain (Gamma Variant) Adapted Immunosensor

**Figure 5:**
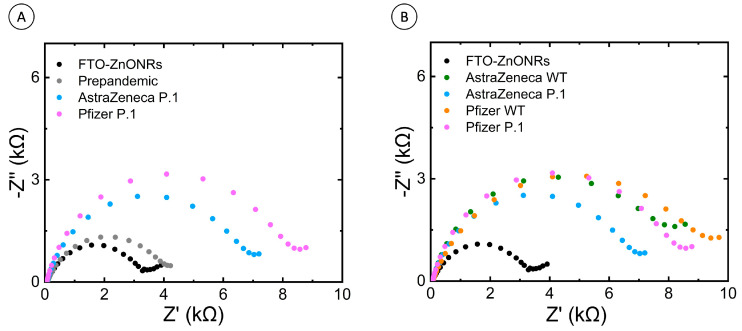
Assessment of polyclonal antibody response in the sera of ChAdOx1-S (AstraZeneca) and BNT162b2 (Pfizer) vaccinated individuals using the FTO-ZnONRs/spike P.1 variant electrode. (**A**) Nyquist diagram of EIS measurements. (**B**) Comparison between responses obtained using the immunosensors FTO-ZnONRs WT and FTO-ZnONRs P.1. Electrolyte: 5 mmol L ^−1^ K_4_Fe(CN)_6_/K_3_Fe(CN)_6_ solution in 0.1 mol L ^−1^ KCl.

**Figure 6:**
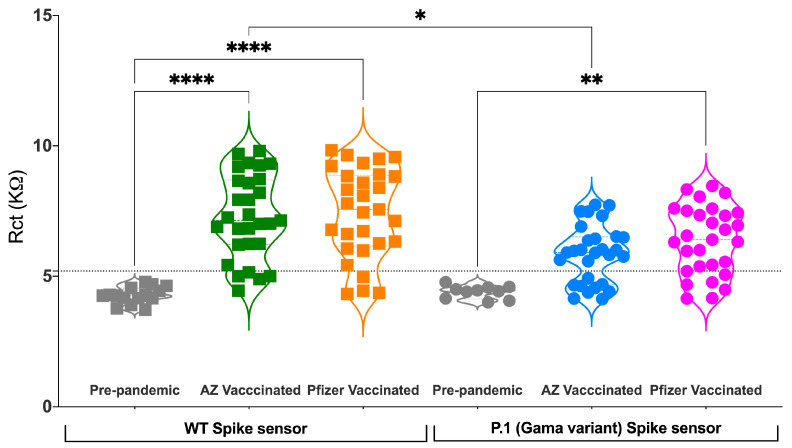
The vaccine-induced polyclonal antibody response performance against S protein from SARS-CoV-2 P.1 (Gamma variant) using the adapted FTO-ZnO nanostructured electrochemical immunosensor. Comparison of FTO-ZnONRs/spike WT and FTO-ZnONRs/spike P.1 immunosensor response between a representative set of clinical samples from spike-based vaccinated individuals. Violin plots, where symbols represent individual participants. Asterisks indicate significant results * *p* < 0.05, ** *p* < 0.01, and **** *p* < 0.0001 using a Kruskal–Wallis test with Dunn’s post-hoc test for multiple comparisons. AZ= ChAdOx1-S (AstraZeneca). Pfizer= BNT162b2 (Pfizer–BioNTech).

The impedimetric response of serum samples from ChAdOx1-S and BNT162b2-vaccinated individuals was performed with the adapted FTO-ZnONRs/spike P.1 immunosensor (Figure 5A). Our results showed that the antibodies induced by immunization with both vaccines designed against the wild-type (WT) strain of SARS-CoV-2 were readily detected by the immunosensor containing the P.1 (Gamma variant) S protein. It is also possible to observe that the R_ct_ signal is lower for the samples evaluated against the P.1 S protein than for those assessed against the viral WT S protein from the original Wuhan isolate (Figure 5B and Figure 6). Several authors have described in the literature a decrease in antibody titers for the P.1 (Gamma variant) compared to the WT [39,40,41]. We also observed that the R_ct_ signal was higher for samples of individuals vaccinated with BNT162b2 than those immunized with ChAdOx1-S (Figure 5B and Figure 6), indicating that the BNT162b2 vaccine induces antibodies with a better capacity to recognize the COVID-19 P.1 VoC. The efficacy of several COVID-19 vaccines against SARS-CoV-2 P.1 (Gamma variant) VoC has already been assessed by clinical case-control studies that evaluated the effectiveness of the BNT162b2 and ChAdOx1-S vaccines or even a large randomized and well-controlled trial (R_CT_) that evaluated only the ChAdOx1-S vaccine. These studies have demonstrated that the BNT162b2 vaccine has slightly superior efficacy when compared to the ChAdOx1-S vaccine for P.1 (Gamma variant) [41]. The fact that we have also captured a subtle difference in serological performance against the S protein from P.1 (Gamma variant) using a modest number of samples indicates the excellent performance achieved by the adapted point-of-care device.

#### 3.2.4. FTO-ZnONRs/Spike WT and FTO-ZnONRs/Spike P.1 Immunosensor Performance Analysis

**Figure 7:**
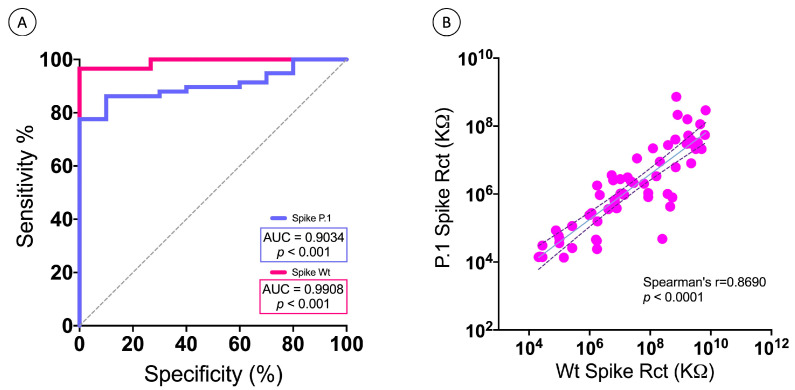
Comparison between the detection accuracy of anti-S antibodies by the developed FTO-ZnONRs/spike WT and the FTO-ZnONRs/spike P.1 immunosensors. (**A**) Receiver Operator Characteristic (ROC) curve comparison between the WT and P.1 immunosensors. (**B**) Correlation between the WT and P.1 immunosensors. Electrolyte: solution of 5 mmol L^−1^ K_4_Fe(CN)_6_ /K_3_Fe(CN)_6_ in 0.1 mol L^−1^ KCl.

The accuracy of the developed electrochemical ZnONRs/spike WT and spike P.1 immunosensors was successfully demonstrated by the comparative received-operating characteristic (ROC) curve analysis shown in Figure 7A. The AUC value of 0.5 in the ROC curve analysis represents random chance, and 1.0 for perfect accuracy [42]. The FTO-ZnONRs/spike WT immunosensor presented an AUC of 0.9908, which was higher than the FTO-ZnONRs/spike P.1 (AUC of 0.9034). Nevertheless, both values indicated the high accuracy of these point-of-care devices in assessing vaccine-induced antibody-mediated immunity. Thus, the developed ZnONRs/spike WT and spike P.1 sensor platforms provide an efficient alternative method for determining anti-SARS-CoV-2 antibody responses in human serum, and have potential applications in estimating the prevalence of the SARS-CoV-2 antibodies in a given population.

#### 3.2.5. Correlation of Anti-S Protein Antibody Levels Results by the WT Spike ZnONRs and P.1 Spike ZnONRs Immunosensors

Correlation between humoral responses against SARS-CoV-2 S protein, analyzed by charge transfer impedance (R_ct_) in serum samples from ChAdOx1-S (AstraZeneca) and BNT162b2 (Pfizer) vaccinated study participants, was performed using the ZnONRs/WT spike and ZnONRs/P.1 spike modified electrode devices. Data from both ZnONRs/spike immunosensors were compared, and the accuracy and Spearman rank correlation were calculated (Figure 7B). A correlation coefficient of 0.8690 was obtained, indicating a highly significant data correlation between the original WT ZnONRs/spike immunosensor and the novel P.1 ZnONRs/spike immunosensor.

## 4. Conclusions

In this work, the ZnO-based biosensing platform has shown potential to evaluate the vaccine-induced humoral immune responses after spike-based COVID-19 vaccination. The electrochemical immunosensor device was validated to detect antibodies against the SARS-CoV-2 WT S protein in serum samples from ChAdOx1-S and BNT162b2 vaccinated individuals with excellent performance. Notably, the adapted SARS-CoV-2 immunosensor for the P.1 (Gamma variant) has successfully discriminated samples from pre-pandemic, BNT162b2 (Pfizer), and ChAdOx1-S (AstraZeneca)-vaccinated individuals. The adapted technology and the FTO-ZnONRs/spike P.1 immunosensor revealed a strong concordance with our group’s previous work based on the wild-type version of the ZnONRs immunosensor [16]. Both WT and P.1 immunosensors presented high accuracy performances with an AUC of 0.9908 and 0.9034, respectively. Data reveals that the adapted immunosensor approach based on S proteins derived from VoCs is promising and might have the potential to be employed as a point-of-care serological testing tool to evaluate vaccine-induced immunity effectiveness for SARS-CoV-2 variants. Furthermore, we demonstrated that the nanostructured modified electrode is modular and shows good potential for serological diagnosis of other diseases.

## Figures and Tables

**Table 1:** Comparison between reported COVID-19 serological tests for detection of antibodies against SARS-CoV-2.

Biorecognition Element	Test Type	Quantitative Range	LOD	References
WT S protein	SPR	0–100 μg·mL^−1^	50 ng·mL^−1^	[31]
WT S protein peptide	OPOCT biosensor	0.012–1 μg·mL^−1^	12.5 ng·mL^−1^	[32]
WT S protein RBD	Paper-based biosensor	1–1000 ng·mL^−1^	0.96 ng·mL^−1^	[33]
WT S protein	Microflow Cytometry	0–1 mg·mL^−1^	100 ng·mL^−1^	[34]
WT S protein RBD	Fluorescence Microscopy		200 ng·mL^−1^	[35]
WT S Protein peptide	DPV technique	0.075–15 μg·mL^−1^	75 ng·mL^−1^	[36]
WT S protein peptide	DPV technique	0.08–5.2 μg·mL^−1^	0.77 μg·mL^−1^	[37]
WT S protein RBD	Chronoamperometry method	0.01–60 μg·mL^−1^	10.1 ng·mL^−1^	[38]
WT S protein	Impedimetric measurements	200–1200 ng·mL^−1^	19.96 ng·mL^−1^	[16]
P.1 S protein	Impedimetric measurements	200–1200 ng·mL^−1^	52.55 ng·mL ^−1^	This work

## Data Availability

The data that support the findings of this study are available from the corresponding author upon reasonable request.

## Abbreviations

The following abbreviations are used in this manuscript:

DPV	Differential pulse voltammetry
FTO	Fluorine-doped tin oxide
OPOCT	Optofluidic point-of-care testing fluorescence
P.1 S protein	SARS-CoV-2 Gamma variant spike protein
SPR	Surface plasmon resonance
S protein	SARS-CoV-2 spike protein
WT S protein	SARS-CoV-2 wild-type spike protein
WT S protein peptide	SARS-CoV-2 wild-type spike protein peptide
WT S protein RBD	SARS-CoV-2 receptor binding domain of wild-type spike protein
ZnONRs	Zinc oxide nanorods
